# Soluble HLA Technology as a Strategy to Evaluate the Impact of HLA Mismatches

**DOI:** 10.1155/2014/246171

**Published:** 2014-09-01

**Authors:** Heike Kunze-Schumacher, Rainer Blasczyk, Christina Bade-Doeding

**Affiliations:** Institute for Transfusion Medicine, Hannover Medical School, Medical Park, Feodor-Lynen-Straße 5, 30625 Hannover, Germany

## Abstract

HLA class I incompatibilities still remain one of the main barriers for unrelated bone marrow transplantation (BMT); hence the molecular understanding of how to mismatch patients and donors and still have successful clinical outcomes will guide towards the future of unrelated BMT. One way to estimate the magnitude of polymorphisms within the PBR is to determine which peptides can be selected by individual HLA alleles and subsequently presented for recognition by T cells. The features (structure, length, and sequence) of different peptides each confer an individual pHLA landscape and thus directly shape the individual immune response. The elution and sequencing of peptides by mass spectrometric analysis enable determining the *bona fide* repertoire of presented peptides for a given allele. This is an effective and simple way to compare the functions of allelic variants and make a first assessment of their degree of permissivity. We describe the methodology used for peptide sequencing and the limitations of peptide prediction tools compared to experimental methods. We highlight the altered peptide features that are observed between allelic variants and the need to discover the altered peptide repertoire in situations of “artificial” graft versus host disease (GvHD) that occur in HLA-specific hypersensitive immune responses to drugs.

## 1. Introduction

Human leucocyte antigen (HLA) class I incompatibilities represent the major barrier for successful outcome in haematopoietic stem cell transplantation (HSCT), a therapeutic strategy for the treatment of hematologic malignancies. The best clinical outcomes for unrelated HSCT can be achieved by an 8/8 match, meaning high resolution matching on HLA-A, -B, -C, and -DRB1 levels [1]. Certain HLA class II mismatches have also gained considerable attention in the selection of unrelated donors; most notably several studies on HLA-DP permissivity have been performed [2–5]. Such a perfect HLA match of a given donor: recipient pair is in most cases impossible to find; therefore, mobilized stem cells from well-matched but unrelated donors are used for transplantation [6]. Despite great care being taken to select the best possible match these transplants may be associated with significant risks of graft versus host disease (GvHD), graft failure, or transplant related mortality [7]. Clinical data have demonstrated that the risk of GvHD correlates with the number of mismatches within the HLA molecule, where both the type of amino acid (AA) substitution and its location can directly influence the degree of histocompatibility [8]. For these reasons, a priority for certain unrelated mismatched donors is also given to those whose polymorphisms do not affect peptide binding. If there are differences between two allelic HLA subtypes, then this is likely to alter their presented peptide binding features; thus alloreactivity is expected [9]. If the peptide binding features are not altered, then alloreactive immune reactions are more unlikely; however, they cannot be fully excluded since the genetic variability in the population may give rise to certain minor histocompatibility antigens. The impact of polymorphisms located within the peptide binding region and the risk of severe acute GvHD is well known [10]; furthermore the residues described to impact the risk of GvHD when mismatched (AA 9, 99, 116, 156) have been directly associated with the peptide anchor p2 or pΩ [11] or indirectly with peptide binding through differential interaction with the peptide loading complex and peptide selection [12, 13].

**Figure 1:**
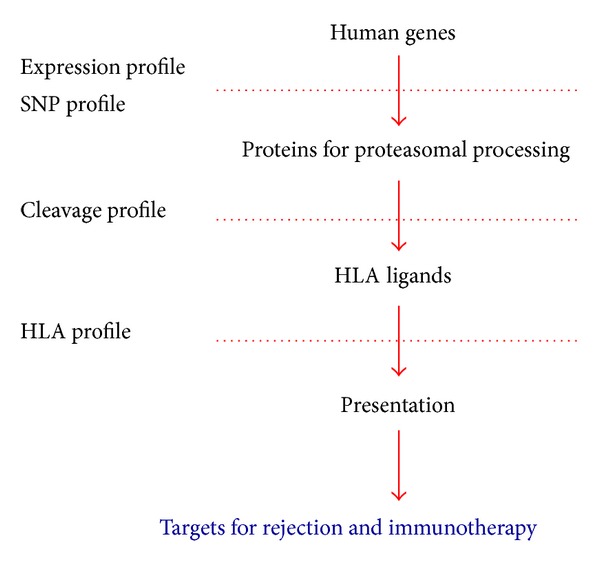
Assessment of targetable allogeneic epitopes. Every ligand has to pass several filters, before being presented in an HLA molecule. The first is the individual expression profile. Proteins then undergo further proteasomal processing, where the individual cleavage profile determines the sequence of possible HLA ligands. The last filter is the individual HLA profile that differs between allelic variants.

The feasibility to find the best acceptable mismatch and have successful clinical outcomes depends on our understanding of the impact of a certain HLA polymorphism. It would be beneficial to have a fast and reliable method that would allow a prediction of individual mismatch magnitudes to help clinicians in deciding which donor: recipient pair has the best chance of successful transplantation. However, the impact of a given mismatch might differ for every single allelic variant and is furthermore affected by the proteomic content that is available for presentation in an HLA molecule, since peptides undergo several selection criteria before being presented in an HLA molecule on the cell surface (Figure 1). Alongside the functional allelic differences conferred by AA exchanges that influence the interaction of the HLA heavy chain with the peptide loading complex, the peptide cargo itself has to fulfill certain criteria. Proteasomally digested peptides are transported into the endoplasmic reticulum via the transporter associated with antigen processing (TAP) and loaded onto immature HLA class I molecules with the assistance of the peptide loading complex (PLC) [14]. Here, it has to be considered that certain HLA alleles select and present their peptides through an alternative pathway, since particular polymorphism can influence which allogenic peptides are selectively bound through conformational alteration of the HLA heavy chain [12]. Peptide selection that did not undergo selection and optimization of the PLC might lead to immunological reactions since it enables the presentation of poorly tolerated peptides. This might therefore mimic foreign proteomic content to immune effector cells and lead to rejection episodes when transplanted.

HLA molecules are highly variable; accepting a mismatched donor means that a comprehensive knowledge should be allocatable. An HLA molecule that is displayed to the immune system consists of three modules, the heavy chain, beta 2 microglobulin (*β*2m), and the individual peptide. While *β*2m is invariant, the heavy chain as well as the peptide is variable, and there are thousands of possible peptide/HLA (pHLA) molecules that can be displayed to immune effector cells. Variations within the AA sequence of the heavy chain may therefore alter substantially the whole pHLA landscape, for that reason the knowledge about the magnitude of a distinct mismatch within the heavy chain is important to understand and/or predict the molecules function. These polymorphisms can affect (i) the individual peptide binding motif [15–18], (ii) peptide selection [12], and/or (iii) the individual pHLA landscape [19, 20] that will be scanned by immune effector cells. Due to the enormous degree of HLA class I polymorphisms (*n*) and the quantity of possible mismatches [magnitude = 1/2 · *n* · (*n* − 1)] that can occur there is still no strategy available for the selection of the less permissive class I mismatch when no identical donor but multiple mismatched donors are available. A mean for a comprehensive measure of histocompatibility can in the context of the extensive polymorphism only take place as a systematic study of key alleles with distinct polymorphism. Certain mismatches as, for example, position 156 in the B*44 allelic group have been studied extensively; clinical data [21] as well as* in vitro* data [12, 22–24] are available. However, for most allelic groups, there is a lack of clinical data or* in vitro* data that could be translated to histocompatibility. To enable a comprehensive and intelligent mismatching strategy, the fast and reproducible analysis of the peptide repertoire is required since it represents the most variable part of a HLA molecule.

## 2. Determination of Mismatch Impact Based on Peptide Sequences

HLA class I allelic peptide motifs have been described for the most frequent variants [26–29], but few studies have addressed the question to which extent variants of the same allelic HLA group differ in their peptide motifs and/or peptide repertoires [30–33].

HLA-B*44:02^Asp156^ and B*44:03^Leu156^ differ by a single AA exchange located in the PBR and are well-studied examples for strong alloreactive immunological episodes. Fleischhauer et al. described in 1990 a strong alloreactive response mediated by T cells that resulted in transplant rejection [21]. To explain this unexpected occurrence, peptide profiles of the mismatched variants have been analysed by the same author in 1994 [22]. Since no differences in peptide profiles could be detected, the unexpected alloreactivity could only be explained in 2003, where Macdonald et al. solved their structures [23] and found subtle alterations in the peptide binding. This example shows the distinct connection between peptide binding profiles and immunogenicity and started a new era of histocompatibility rating.

The nature of a given AA mismatch plays a significant role in deciding for histocompatibility; in that regard, it is thought that AA exchanges from the same chemical group are more permissive than those AAs from different chemical groups. The allelic variants B*44:02^Asp156^ and B*44:35^Glu156^ differ at a single mismatch; both of the AAs exchanged are polar acidic and thus not expected to confer alteration of the PBR feature. However, the comparison of the peptides from B*44:02^Asp156^ and B*44:35^Glu156^ showed an unexpected alteration in the binding motif of the peptide's pΩ [34]. B*44:35^Glu156^ was found to bind and present a significantly high number of peptides of extraordinary length and to disfavour the binding and presentation of canonical peptides. The peptide data derived from B*44:35^Glu156^ highlighted a strong shift in the binding preference for Lys at the C-terminus, while this residue was completely absent in peptides eluted from B*44:02^Asp156^. The observation of such a change in the peptide anchor in B*44:35^Glu156^ as a result of a highly conserved substitution at position 156 was unexpected. Based on the structure of B*44:02^Asp156^ (PDB 1M6O) [23] we were able to model B*44:35^Glu156^ bound to a peptide with the distinct binding motif and therefore explain the structural alteration. Cellular studies using alloreactive T cells supported the findings of the peptide diversities. In this case, the preliminary peptide data were essential to explain the strong T-cell mediated alloreactivity.

Further studies defined the differential impact of single mismatches on peptide binding and the paradox that an accumulation of multiple polymorphism located in the PBR does not alter the designated peptide binding motif.

Most class I alleles show the highest sequence polymorphism in the α1 and/or α2 domain that form the PBR and are encoded by exons 2 and 3, which therefore constitute the region of interest for histocompatibility matching. Currently, allelic variants with mismatches outside of the α1 or α2 domains are thought to have the same immune function and thus would represent a permissive mismatch in HSCT. HLA-B*44:02:01:01 and B*44:27 are considered to be functionally identical since they differ by the micropolymorphism Val199Ala, located in the α3 domain. To validate the theory that B*44:02^199Val^ and B*44:27^199Ala^ represent functionally identical alleles, we compared peptides and their features derived from both B*44:02^199Val^ and B*44:27^199Ala^. The mismatch at residue 199 did not alter significantly the peptide motif, the peptide features, nor the peptide repertoire [35]; for that reason a single 199 mismatch for B*44 variants might be considered as a permissive mismatch, but must be certainly validated by cellular assays. According to our finding Bettens et al. investigated in 2013 the alloreactivity of CD8+/CD137+ T cells in a mixed lymphocyte culture reaction and could demonstrate a complete lack of allorecognition between the Val199Ala mismatched variants [36]. These findings supported our conclusion that in the case of B*44:02:01:01/B*44:27 incompatibility, we could define histocompatibility in terms of the features of the bound peptides.

**Figure 2:**
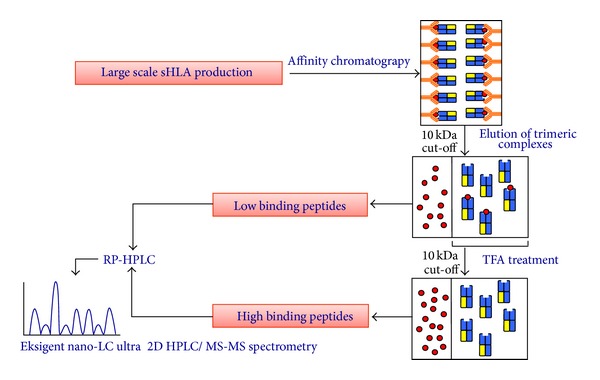
Generation of peptide sequences from sHLA molecules. This figure gives a schematic overview of the steps towards the generation of peptide sequences obtained from sHLA molecules. The HLA heavy chain is given in blue; *β*2m is shown in yellow; and the peptide is shown in red. Cell culture supernatants containing sHLA molecules are passed over an N-hydroxysuccimide- (NHS-) activated HiTrap column coupled to mAb w6/32. Trimeric complexes (class I heavy chain, *β*2m and peptide) are eluted using a pH 2.7 elution buffer. Here, peptides from sHLA complexes can be differentiated into low and high binding peptides. The trimeric elution fractions are filtered through a 10 kDa cut-off membrane and the peptides detected in the flow through are considered to be of low affinity. The retentate containing dimeric (heavy chain and *β*2m) as well as trimeric complexes is then treated with 0.1% trifluoroacetic acid (TFA) to elute high binding peptides that can finally be separated by filtration through an additional 10 kDa cut-off membrane. Flow through fractions containing the low or high affinity peptides are subjected to mass spectrometric analysis using an Eksigent NanoLC Ultra 2D HPLC coupled to an orbitrap ion trap. Database queries can finally be carried out using Mascot software [25] incorporating the IPI human and the respective decoy databases.

The knowledge of allele specific peptide binding data enables an isolated and individual assessment of every AA mismatch. Through comparative analysis of the peptides derived from HLA-B*44:02 and B*44:08, differing at residues 41, 45, and 46, we were able to exclusively assign position 45 of the heavy chain to significantly affect the peptide binding motif while residues 41 and 46 had no influence on the bound peptides [16]. While HLA-B*44:02 has a peptide anchor motif of Glu at p2, B*44:08 binds at p2 Gln and Leu. The peptide data provide evidence that mismatches at position 45 for B*44 variants should be avoided, since it could be assumed that an alteration of the peptide features would most likely lead to a conversion of the accessible pHLA surface and consequently affect T-cell recognition. The comparison of peptide data for, HLA-B*44:02 and B*44:06, both of these subtypes are being distinguished by 7 residues polymorphic (24, 32, 41, 45, 63, 67, and 80), showed a differential impact on peptide binding and allowed for assignment of each residue within the heavy chain to a certain residue within the peptide [11]. Residues 45, 63, and 67 are highly polymorphic and show in all pHLA structures analysed abundant contact frequencies to the anchoring p1 and p2 position of peptides [11], highlighting their role in peptide specificity. By soluble HLA technology we could demonstrate the differences in peptide anchoring. While B*44:02 is not anchored by p1, but by Glu at p2 and Phe, Tyr, and Trp at the C-term, B*44:06 is anchored by Asp and Glu at p1, Pro and Ala at p2, and Tyr and Trp at the C-term. Moreover, MALDI-TOF analysis (a tool for the mass analysis of peptides) of low or high affinity peptide pools (Figure 2) suggest that in contrast to B*44:02, B*44:06 preferably selects high affinity peptides and binds peptides tightly. Additionally, B*44:02 had a tendency to bind extraordinary long peptides whereas peptides bound to B*44:06 were restricted to the canonical length of 8–10 AAs. The knowledge of peptide data for B*44:06 allowed detailed structural investigation of the mismatch impact. Therefore we could demonstrate that in both alleles Ser167 allows the nonpolymorphic Arg170 to hydrogen bond with the peptide p1 residue. For B*44:06, molecular modeling suggests that the Glu63 > Asn63 polymorphism enhances the preference of the P1 pocket for acidic AAs [11]. Translating these observations into histocompatibility, residue 63 should be considered as a nonpermissive mismatch. This is further supported by the observation that its frequency in contacting the peptide at main positions (p1, p2) could be observed in >95% of pHLA structures reflecting a distinct constraint of peptide motifs for B44 variants [11]. This data allowed “alloreactivity ranking” of the mismatched residues within the B*44:06 heavy chain.

Peptide data from HLA-B*44:09 showed how the 5 polymorphic residues (77, 80, 81, 82, and 83) distinguishing B*44:09 from B*44:02 affected the peptide binding motif. Both alleles illustrate an E at p2. In contrast to the C-terminal peptide binding motif of B*44:02, Trp, Phe, Tyr, or Leu, B*44:09 derived peptides are restricted predominantly to Leu or Phe. A small percentage of peptides were shared between the allotypes, those peptides contained the restricted B*44:09 anchor motif of Phe or 10 Leu at the pΩ position [17]. The modeled structure of B*44:09 reveals that residues 77 and 80 contact the peptide main chain at the peptide's pΩ; however, residue 81 contacts the peptide's pΩ side chain and appears to be the main cause of sequence specificity at the peptide's C-term. Similarly as described for B*44:06, residue 81 in B*44:09 could be demonstrated to have a high impact on peptide specificity.

It could also be demonstrated that certain alleles sharing the same peptide motif with their mismatched allelic variant show a higher affinity for peptides of an extraordinary length, an observation that is only possible through extended peptide sequencing. This phenomenon could be demonstrated for peptides bound to subtypes of the HLA-B*41 group, HLA-B*41:03, and B*41:04 [20]. Further examination of these variants bound to previous sequenced peptides by high resolution X-ray crystallography demonstrated that polymorphism at positions 97 and 114 in B*41 variants have a strong impact on the steric and electrostatic properties of the PBR and explained their divergent peptide confirmations.

## 3. Individual Peptide Repertoires Explain the Basis of GvHD-Like Drug-Mediated Hypersensitivity Reactions

Another example how a single mismatch within the HLA heavy chain influences the immunological self and guides towards biased T-cell responses is the alteration of presented peptides through the interference of small drug molecules, resulting in the phenomena of HLA-associated drug hypersensitivity. Here, a single AA mismatch within the PBR might change the susceptibility of an allele to allow a small drug molecule to bind and modulate the PBR as it could be demonstrated for the abacavir sensitive allele HLA-B*57:01 [37]. Here, abacavir is able to occupy part of the PBR and modifies the selected peptide repertoire thereby causing a strong T-cell-mediated immune response that is resolved upon withdrawal of medication.

For the carbamazepine sensitive allelic variants HLA-A*31:01 and -B*15:02 we could recently demonstrate distinct functional differences through sequencing of their bound peptides (Kunze-Schumacher et al., 2014, manuscript in preparation).

Understanding the mechanistic basis of drug-mediated hypersensitivity reactions can only take place as a measurement of the allele specific individual peptide repertoire.

## 4. Soluble HLA Technology

Peptide sequencing by the use of soluble HLA (sHLA) technology is a powerful and convenient tool towards the understanding of histocompatibility and high throughput screening of the individual allele specific immunopeptidome. Another possibility to obtain peptides is the classical way of isolating membrane bound HLA molecules from cells; however, this method necessitates high numbers of cellular material. Besides, treatment of cells using lysis buffers might lead to failure of obtaining low affinity peptides, a problem that is not of any concern when using sHLA technology. It could be demonstrated that peptides obtained by soluble technology show the same repertoire as peptides obtained from membrane bound molecules [38]. Furthermore sHLA molecules select their peptides through the same loading pathway and are associated with the peptide loading complex in the same way than membrane bound molecules [12]. Soluble HLA technology thus represents a simple and easy instrument for the determination of mismatch magnitude.

For the use of sHLA molecules exon 1 through 4 from a given HLA variant is cloned into an appropriate expression vector and transferred preferentially into an HLA class I negative cell line, for example, LCL 721.221 cells. When the question has to be answered is what peptides are presented during a viral infection or in certain tumor cell, it is as well possible to clone a recombinant C-terminal tag (e.g., V5, c-Myc) for further purification, which would enable the exclusive purification of the desired recombinant HLA molecule without contaminations of native HLA molecules. The highest rate of sHLA expressing cells is achieved through lentiviral transduction of the desired HLA subtype in target cells as described previously [12]. For large scale sHLA production cells are transferred into bioreactors and sHLA molecules can be directly purified from the cell culture supernatant using an appropriate antibody, for example, W6/32 (Figure 2) bound to an affinity column. Peptides can be directly isolated from trimeric sHLA complexes and differentiated into low or high affinity peptides.

Soluble HLA technology offers the flexibility to achieve high amounts of proteins that are secreted in the cell culture supernatant without the need of high cell numbers. To quantify the protein concentration a sandwich enzyme-linked immunosorbent assay (ELISA) can be used as a simple and easy method for high throughput screening. Here, the anti-HLA-A-B-C W6/32 [39, 40] monoclonal antibody is employed as capture antibody; HRP-conjugated anti-*β*2m mAb serves as the detection antibody [12].

## 5. Peptide Data Are the Basis for Bioinformatic Prediction Tools

Numerous peptide binding prediction algorithms are available; they can be categorised into three major groups: motif and scoring matrix based methods, hidden Markov models, and artificial neural networks [41–45]. However, all those prediction algorithms are based on the limited knowledge of available peptide binding data that means prediction is only possible for a small fraction of known MHC proteins. The growing number of HLA alleles makes it impossible to provide prediction tools for variants or certain polymorphism where no experimental data exist. Structure based methods for predicting the allogenicity of a certain mismatch can only take place for alleles with known peptide sequences. Experimental peptide binding data and allele specific peptide motifs allow for the prediction and ranking of T-cell epitopes by certain prediction algorithms such as SYFPETHI [46, 47], NetMHC [48], RANKPEP [49], PeptideCheck, [50] or BIMAS [51]. Here it has to be taken into account that* in silico* peptide binding prediction such as for vaccine development can only take place when peptide sequences and binding specificities for an allele of interest have been identified previously. If the allele specific peptide binding features, the motif, or the preferred length is known, it is possible to predict peptides and peptide binding data from desired proteins such as virus epitopes, which might be tested by* in vitro* systems [52–54]. However, all these programs are not able to address the binding and presentation of noncanonical peptides (>8–10 AAs) [55] that might elicit strong alloreactive T-cell responses [56, 57] and it is well known to date that certain HLA variants bind peptides of unusual length [20, 58, 59]. Several long peptides for HLA class I molecules have been predicted by extending known shorter epitopes or by screening peptide libraries, including overlapping 16-mer [60] or 15-mer peptides [61, 62]. Even the binding of peptides with a length up to 25 AAs could be observed [58], suggesting that the length limitation of naturally processed HLA bound peptides is primarily controlled by their availability following antigen processing. The knowledge of the allele specific self peptidome will help to update current programs and bypass these limitations.

The prerequisite for comprehensive prediction tools is the knowledge about functional differences of HLA key alleles. Two of these tools, namely,* histocheck* [63] and* pocketcheck *[11], will be explained in the following.* Histocheck* is a tool to support an estimation of the allogenic potential between mismatched HLA variants. The rating score between two mismatched variants is based on the dissimilarity of the mismatched AAs. Furthermore,* Histocheck* designates and visualizes TCR and/or peptide binding AAs and directs towards estimation of histocompatibility. These data are based on available structures of pHLA complexes adapted from the classical pocket definitions, specified in 1991 by Saper et al. [64] and updated in 1996 by Chelvanayagam [65]. There have been several attempts to define which positions in the HLA binding groove influence the specificity of bound AAs at each position in the peptide based on X-ray crystallography. Structure databases (e.g., RCSB protein data bank) are the basis for molecular modeling of alleles whose structures have not been solved by X-ray crystallography, yet [16, 17], but have been investigated on the basis of their presented peptides. The generation of these structural data is only possible in the context of known allele specific peptides. Over the years more than 100 crystal structures became available that facilitated a broader definition of pockets,* pocketcheck* [11]. X-ray crystallographic determination of pHLA class I complexes provided valuable information for understanding how peptides bind to individual HLA class I molecules. Precisely, the allogenicity of a given mismatch is based on the information which residues within the HLA PBR would contact which peptide residue.

Several attempts have been taken to predict peptide binding to MHC molecules [49, 51, 66–71] for the assessment of permissivity or for vaccine development. For cellular therapeutics, CTLs that target infected cells require the identification of epitopes that distinguish healthy and infected cells. The knowledge about the self-proteomic content is therefore imperative for comprehensive and updated prediction tools.

If predicted epitopes of pathogenic origin would ever be presented* in vivo* remains questionable, before they have not been isolated and determined. Therefore “native” peptide data represent the prerequisite for all further prediction tools.

Successful prediction programs may significantly enlarge the donor pool for cellular, peptide based vaccination therapies and for certain patients by enabling a ranking of mismatches, building a bridge towards intelligent mismatching for better clinical outcomes.

## 6. Conclusion

The knowledge about the impact of distinct HLA class I mismatches in the context of experimental data such as peptide data and functional T-cell studies along with clinical data, if available, is appreciated by the feasibility to mismatch patients and still have successful clinical outcomes. Our approach in dividing peptides generated by sHLA technology into low or high affinity also allows a prediction of the relative half-life time and thus relative immunogenicity of a given pHLA complex.

Given the fact that every HLA allele has an individual peptide repertoire, it becomes obvious how many single pHLA molecules are available for an antigenic T cell. For that reason it has been proposed that it should be assessed whether GvHD and GvL can be related to the recipient specific peptide repertoire and its set of shared peptides by the donor HLA molecule [72]. While the risks associated with HLA-mismatches for unrelated bone marrow transplantation are clear, it becomes important to understand how certain peptides are selected by distinct alleles and thus how a given mismatch can influence the peptide selection and presentation process.

Towards that line, soluble HLA technology is a simple and powerful instrument for the detection of peptide binding motifs from allelic HLA variants. As a tool for high throughput analysis of polymorphism on HLA function, peptide sequencing enables evaluation of the best match and to highlight the potential risks of unmatched transplantation. Furthermore, peptide data represent the prerequisites for successful antitumor therapies. However, the high cost of peptide sequencing limits the acquisition of binding data. For that reason, it is imperative to select key alleles that enable the prediction of different allelic subtypes. Peptide motif based ranking of allelic mismatches can contribute to the donor selection process when no HLA-identical donor is available to prevent mismatching from being a matter of chance.

## Abbreviations

HLA: Human leucocyte antigen
HSCT: Hematopoietic stem cell transplantation
GvHD: Graft versus host disease
pHLA: Peptide-HLA complex
PBR: Peptide binding region
AA: Amino acid.
